# Lifetime alcohol‐use prevalence and correlated factors among street children in Iran

**DOI:** 10.1002/brb3.2781

**Published:** 2022-10-13

**Authors:** Payam Roshanfekr, Delaram Ali, Mehdi Noroozi, Giti Bahrami, Meroe Vameghi

**Affiliations:** ^1^ Social Welfare Management Research Center University of Social Welfare and Rehabilitation Sciences Tehran Iran; ^2^ Social Determinants of Health Research Center University of Social Welfare and Rehabilitation Sciences Tehran Iran; ^3^ Social Determinants of Health Research Center Alborz University of Medical Sciences Karaj Iran

**Keywords:** alcohol use, Iran, lifetime, street children

## Abstract

**Background:**

Several studies on street children in Iran reported a high prevalence of alcohol consumption among this group. This study assessed the prevalence of lifetime alcohol use and correlated factors among street children in Iran.

**Methods:**

We conducted a cross‐sectional survey among 856 street children from six provinces of Iran. Behavioral data were collected by trained interviewers using a structured questionnaire. Our target outcome was lifetime alcohol use. We examined associations between individual variables and lifetime alcohol use using the chi‐square. A multiple logistic regression model included variables with a *p*‐value < .2. Lastly, we reported the adjusted odds ratio (an OR) point estimate and 95% confidence interval (95% CI) as the effect measure.

**Results:**

Mean age and standard deviation (SD) of alcohol drinkers were 14.94 ± 2.16. Overall, 16.6% (CI95%: 14.38%, 19.55%) of participants reported lifetime alcohol use, and almost 60% of children reported alcohol use over three past months. In the final model, factors that were independently associated with alcohol use included the 15–18 age range (AOR 2.35, 95% CI 1.48−3.73), Iranian nationality (AOR 3.36, 95% CI 2.07−5.45), working longer than 5 years in the streets (AOR 2.90, 95% CI 1.72−4.88), father's drug use (AOR 1.93, 95% CI 1.22−3.01), and illiteracy (AOR 1.65, 95% CI 1.03−2.66).

**Conclusions:**

The results of the present study demonstrated that preventive plans for alcohol use among street children must be addressed using the services provided by governmental and nongovernmental organizations.

## Graphical abstract

• Findings suggest an increase in alcohol consumption among the street children in recent years.

• The rate of alcohol consumption might have been under‐reported by street children of Iran, due to legal prohibition of alcohol use.

• There were no differences in alcohol consumption between males and females.

• Iranian nationality is associated with an increased risk of alcohol consumption in comparison to Afghan nationality.

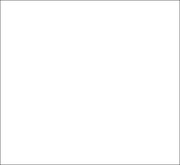

## 1 INTRODUCTION

Alcohol use among adolescents is a global concern. As reported by World Health Organization (WHO), more than a quarter (26.5%) of all 15−19‐year‐olds are current drinkers worldwide. Prevalence rates of current drinking are highest in the WHO European Region (43.8%), the Region of the Americas (38.2%), and the Western Pacific region (37.9%) (WHO, 2019). Besides, in many countries of the three regions, alcohol consumption starts before 15 years and could be 50–70% among 15‐years‐old people (WHO, 2019). Similarly, a recent systematic review in India has indicated that lifetime alcohol consumption among adolescents could be 69.8% (Nadkarni et al., 2022).

Several studies were conducted on the bio‐behavioral characteristics of street children as a vulnerable group of adolescents and reported a significant prevalence of high‐risk behaviors, such as drug and alcohol use in this group. A systematic review of the epidemiology of substance use in 22 countries in 2014 showed that alcohol is the most commonly used drug after inhalants and tobacco in street children and youth (Embleton et al., 2013). Meanwhile, different prevalences of alcohol abuse among street children have been reported in different places and times. Although an investigation in Ethiopia reported 55.8% alcohol use among street children on a daily basis (Buruh et al.), a more recent study in Ethiopia (2019) showed that 11.4% of children used alcohol all the time (Ayenew et al., 2020). Another study in India (*N* = 603) indicated that 42% of street children had a history of alcohol consumption, 4% of whom consumed alcohol daily, and 26% used weekly (Reddy et al., 2014). A study on street children living in Ghana (*N* = 227) reported lifetime and daily consumption of alcohol use to be 81.3% and 16.2% among children, respectively (Asante et al., 2014).

Other investigations have highlighted the harmful consequences of alcohol consumption in individuals aged below 18 years. A research study conducted in the United Kingdom argued that after alcohol consumption, street children faced problems with the police three times higher, fought five times more, and injured themselves 14 times higher than in normal circumstances (Fuller & Hawkins, 2012). In addition, alcohol consumption is recognized among determinant factors of high‐risk behaviors. Research in Nepal suggested that alcohol consumption in street children is a determinant characteristic of active substance use (AOR  =  2.66; 95% CI  =  1.46−4.84) (Kakchapati et al., 2018).

Some studies on street children in Iran also reported a high prevalence of alcohol consumption among this group. For instance, a study in Tehran (*N* = 579) documented that alcohol consumption among street children was 7.8% (Khaniha et al., 2014). Another investigation on street children (*N* = 289) in Tehran (2015) reported that 17.3% consumed alcohol at least once throughout their lifetime, and 7% reported alcohol consumption daily. Moreover, the same study suggested that 57.6% of the children with at least one‐time substance abuse consumed alcohol every day (Dejman et al., 2015).

Studies conducted in other Iranian cities revealed alcohol consumption among street children. For example, a study reported a 2% alcohol consumption prevalence in street children in Isfahan (*N* = 386) (Ataei et al., 2010). Another research documented that the prevalence of alcohol consumption was 2% among street children in Zahedan (Ansari et al., 2016).

The literature review on street children's bio‐behaviors indicated that most studies have mainly focused on Tehran as the capital city. However, studies in other Iranian cities disclosed diverse results in terms of alcohol consumption among street children. The present study is the first large‐scale attempt at alcohol consumption prevalence across six cities in Iran to address the data gap between Tehran and other cities.

## 2 MATERIAL AND METHODS

We conducted a cross‐sectional survey among 856(response rate 98.5%) street children from the center of six provinces in Iran, including Mashhad, Karaj, Kermanshah, Bandar‐abbas, Zahedan, and Tehran (the capital city), from March to May 2017. We selected cities based on geographical and ethnic diversity to consider cultural variation. Study participants were recruited using the TLS method. Eligible individuals aged between 10 and 18 years old were working or living at least a few hours each day for at least a preceding month in the streets and gave informed consent to complete the interview. The interviews were conducted in working venues for street children, such as street intersections, parks, metro stations, and shopping malls. Before gathering behavioral information, we created a list of street working venues and specified the maximum number of children in terms of days and times in each venue in the cities according to interviews. We then completed the list in a group discussion with street children. Then, we undertook random sampling for the venues in the list and confirmed children's presence in the selected venues. The study method and sampling are completely described elsewhere (Ghasemi et al., 2019).

Behavioral data were collected by trained interviewers using a structured questionnaire. Interviewers were staff members of a nongovernmental organization, which provided services for street children in each city, and were trained in a 3‐day workshop held by researchers in Tehran. The questionnaire included modules on sociodemographic characteristics, sex (boy vs. girl), age (years), nationality (Immigrant/refugee, Iranian), educational level (Going to school, Out of school), drug use history (yes, no), age of street work initiation, alcohol use history (yes, no), father's education level (Illiterate, Literate), father's employment status (employed, unemployed, dead), and father's drug use history (yes, no). Our expected outcome was *Lifetime alcohol use* (no vs. yes), which was defined as alcohol use history in life and was assessed on the basis of the following question: “Have you ever used alcohol?.” The answers were coded as “1 = yes and 0 = no.” First, we examined associations between individual variables and *Lifetime alcohol use* using the chi‐square. A multiple logistic regression model included the variables with a *p*‐value <.2. Lastly, we reported the adjusted odds ratio (an OR) point estimate and 95% confidence interval (95% CI) as the effect measure.

## 3 RESULTS

For the analysis, we included 843 street children who provided information on alcohol use. In Table 1, we compare the characteristics of children with alcohol use history in terms of sex, age, education, nationality, working years on the streets, self‐reported lifetime drug use, as well as father's education, employment status, and drug use.

**TABLE 1:** Population characteristics and odds ratios (AOR) of risk factors associated with lifetime alcohol use among street children in Iran, 2016

Variables(determinants)	Lifetime alcohol use			OR	*p*‐Value
	Used *N* (%)	Nonused *N* (%)	Total *N* (%)		
**Sex**					
Girl	13(9.2)	71(10.2)	84 (Khaniha et al., 2014)	1	
Boy	128(90.8)	626(89.8)	754(90)	1.11	.724
**Age** (mean + SD)					
10–14 years	58(40.8)	454(46.8)	512(60.7)	1	
15–18 years	84(59.2)	247(35.2)	331(39.3)	2.66	.000
**School**					
Going to school	59(43.1)	365(52.7)	424(51.1)	1	
Out of school	78(56.9)	327(47.3)	405(48.9)	1.47	.039
**Nationality**					
Immigrant/refugee	34(23.9)	411(58.6)	445(52.8)	1	
Iranian	108(76.1)	290(41.4)	398(47.2)	4.50	.000
**Father's education**					
Illiterate	52(36.9)	327(46.8)	379(45.2)	1	
Literate	89(63.1)	371(53.2)	460(54.8)	1.5	.031
**Fathers job**					
Have income(employed)	23(53.5)	450(60)	473(59.6)	1	
Unemployed	13(30.2)	223(29.7)	236(29.8)	1.17	.761
Death	7(16.3)	77(10.3)	84(10.6)	1.72	.319
**Father's history of drug use**					
non user	62(50.0)	449(72.4)	511(68.7)	1	
User/ex user	62(50.0)	171(27.6)	233(31.3)	2.62	.000
**Duration of child work in street**					
under 3 years	50(35.7)	394(57.2)	444(53.6)		
3–5 years	33(23.6)	159(23.1)	192(23.2)	1.63	.043
More than 5 years	57(40.7)	136(19.7)	193(23.3)	3.30	.000
**Lifetime drug use**					
No	112(94.7)	653(97.9)	765(79.4)	1	
Yes	29(5.3)	14(2.1)	43(20.6)	12.07	.000
**Place of work (city)**					
Tehran	55(38.7)	343(48.9)	398(47.2)	1	
Mashhad	28(19.7)	172(24.5)	200(23.7)	1.01	.952
Karaj	19(13.4)	71(10.1)	90(10.7)	1.66	.084
Kermanshah	25(17.6)	38(5.4)	63(7.5)	4.10	.000
Zahedan	6(4.2)	40(5.7)	46(5.5)	.93	.885
Bandarabas	9(6.3)	37(5.3)	46(5.5)	1.51	.605

The mean age and standard deviation (SD) of alcohol drinkers were 14.94 ± 2.16. Overall, 16.6% (95%CI: 14.38%, 19.55%) of participants reported lifetime alcohol use, and almost 60% of children with alcohol consumption reported alcohol use in the last 3 months. The results of the univariate logistic regression analyses are shown in Table 1. Children's age, educational status, nationality, street work years, drug use history, and their father's drug use history and education were associated with alcohol use among street children.

Children who have reported alcohol use were 15–18 years old (59.2% vs. 40.8%, *p* = .00), were out of school (56.9% vs. 43.1%, *p* = .04), had Iranian nationality (76.1% vs. 23.9%, *p* = .00), and reported more than 5 years of working in the streets (59.3% vs, 40.7%, *p* < .0). Among the children with lifetime alcohol use, 17.6% were from Kermanshah city. Based on the odds ratio (OR = 4.1, *p*‐value .000), the probability of alcohol consumption among street children in this city is 4.1 times more than in Tehran. Drug use history among children who used alcohol was significantly less than those who did not (5.3% vs. 94.7%, *p* < 0).

Table 2 shows the multivariate logistic model of variables associated with alcohol use among street children. In this model, statistically significant (*p* < .2) factors in univariate analyses were included in the multivariate model. In the final model, factors that were independently associated with alcohol use included age range of 15–18 years (AOR 2.35, 95% CI 1.48−3.73), Iranian nationality (AOR 3.36, 95% CI 2.07−5.45), more than 5 years working in the streets (AOR 2.90, 95% CI 1.72−4.88), father's drug use history (AOR 1.93, 95% CI 1.22, 3.01), and father's illiteracy (AOR 1.65, 95% CI 1.03, 2.66).

**TABLE 2:** Adjusted odds ratios (AOR) of risk factors associated with lifetime alcohol use among street children in Iran, 2016

Variables	Lifetime alcohol use		
	AOR	95%CI	*p*‐Value
**Age** (10–14[r] vs. 15–18)	2.35	1.48, 3.73	.000
**Educational status** (Going to School[r] vs. Out of school)	1.22	0.78, 1.90	.377
**Nationality** (immigrant/refugee[r] vs. Iranian)	3.36	2.07, 5.45	.000
**Father's history of drug use**(Non user[r] vs. User/ ex user)	1.92	1.22, 3.01	.004
**Duration of street work**			
under 3 years[r]	1		
3–5 years	1.30	0.73, 2.31	.356
More than 5 years	2.90	1.72, 4.89	.000
**Father's education** (illiterate[r] vs. literate)	1.65	1.03, 2.66	.037
Constant	0.22	0.01, 0.04	.000

## 4 DISCUSSION

The current study examined the status of alcohol consumption and its affiliates among 843 street children in Tehran, the capital city of Iran, and five Iranian cities. Based on our results, 16.6% of street children in the six cities reported alcohol consumption, which ranged from 12.8% in Zahedan to 39.1% in Kermanshah. Also, 14% of street children in Tehran reported alcohol consumption. In contrast with our findings, a few studies reported less alcohol consumption prevalences with different rates among street children across Iranian cities. For example, the prevalence rates in Tehran (Dejman et al., 2015; Khaniha et al., 2014) were 7.8% and 11% in 2008 and 2010, respectively. Two other studies reported alcohol consumption history as 5.2% in Isfahan (Ataei et al., 2010) and 2.3% in Zahedan (Ansari et al., 2016). Only one study reported a 40.1% of alcohol consumption prevalence in Tehran (Shoghli & Mohrez, 2010), though half of the children in their study worked in places other than the streets. There are no national surveys on alcohol consumption among children and adolescents in Iran. Studies on adults also reported different prevalence rates for alcohol with no specific trends. The Ministry of Health and Medical Education of Iran indicated that alcohol was the most prevalent substance in Iran in 2012, with an incidence rate of 5% among the youth a year earlier (Eskandarieh et al., 2013). A recent study also estimated alcohol consumption to be 2.3% in an urban population above 15 years old (Nikfarjam et al., 2017). A systematic review of people under 18 years old (*N* = 80588) reported alcohol consumption equal to 14.7% among Iranian adolescents (Ansari et al., 2016). According to another systematic review on high‐school students in Iran, alcohol was the most prevalent substance and abused by at least 9.9% of the participants (Momtazi & Rawson, 2010). The prevalence rate of alcohol consumption was 10.8% in high‐school students of Yazd, Iran (2017). Overall, alcohol consumption seems higher compared to other substances among young populations in Iran. It could be noted that the high alcohol consumption rate among street children indicates its prevalence all over the country. Also, the higher rates obtained by the present study compared to earlier investigations suggest an increase in alcohol consumption among street children in recent years.

In general, alcohol, cigarettes, and Hashish are the most frequently used substances among street children worldwide (Embleton et al., 2013). Studies in other countries reported higher alcohol consumption rates compared to Iran. Alcohol consumption was reported among street children as 63.2% in Colombo of Sri Lanka (Senaratna & Wijewardana, 2012), 64.3% in Nepal (Kakchapati et al., 2018), 81.3% in Ghana (Asante et al., 2014), 69% in Nigeria (Olley, 2006), and present alcohol consumption of 35% in Egypt (Nada & El Daw, 2010). Alcohol prohibition based on religion and law may decrease alcohol abuse by street children in Iran. However, alcohol consumption might have been underreported by street children in our study due to legal prohibition and penalties. Furthermore, as the literature shows, most street children in Iran have a family (Vameghi et al., 2011), which could be a protective factor against drug and alcohol use. On the other hand, a high percentage of young children in our sample (60%) with low alcohol use may affect total alcohol abuse.

The results suggested that alcohol consumption increased by 2.3 in the age group of 15–18 years. Moreover, living on the streets for more than 5 years is also associated with a triple risk for alcohol consumption. According to a systematic review of worldwide studies, both variables are correlated with alcohol consumption among street children (Embleton et al., 2013). Long working and living in the streets and less family protection for older children could increasingly attract children to street culture with dominant drug and alcohol abuse. Besides, our findings showed high alchol use among children who had fathers with a drug use history. These children may experience less protection for their drug and alcohol use as a result of fathers' absence due to legal problems or less concern about children's alcohol use.

Our findings showed no differences between boys and girls in alcohol consumption, which is similar to two other studies in Iran by Ahmadkhaniha and Dejman (Dejman et al., 2015; Khaniha et al., 2014). However, Ataei et al. (2010) in Isfahan reported a higher prevalence (5.2%) of alcohol consumption among female street children compared to males (1.9%). In general, girls seem to have at least the same rate of alcohol consumption as males in Iranian studies. Some investigations in other countries have documented a higher prevalence of alcohol and cigarette consumption compared to other substances in females. For instance, A study in Accra reported 90.1% and 73.7% alcohol consumption in females and males, respectively. In addition, cocaine, glue, and heroin abuses in females were twice as much in males (Asante et al., 2014). However, current alcohol use was higher in males of all age groups compared to females in Alexandria and Cairo (Nada & El Daw, 2010).

Among the cities in our study, Kermanshah had the highest prevalence rate of alcohol consumption among street children, with a significant difference from other cities. Kermanshah was the only city where no child from Afghanistan participated in the study due to certain prohibitions for Afghan immigrants. According to the data, Iranian nationality was associated with an increased risk of alcohol consumption up to 3.36 times, which could partially explain high alcohol consumption among street children in Kermanshah. Similarly, a previous study in Tehran also documented a lower prevalence of alcohol consumption among Afghan street children in Iran (Dejman et al., 2015). In addition, approximately one‐third of street children from Kermanshah were gypsies (Ghasemi et al., 2019), and alcohol and substance use is a common practice in the families of such children (Vameghi et al., 2011).

A qualitative study in Tehran also indicated that alcohol and substance abuse were generally lower among Afghan street children and their families compared to the Iranians. Based on these findings, the families of Afghan street children living in Iran have more control over their children in terms of alcohol and substance abuse and other social issues and are more conservative due to living as migrants (Dejman et al., 2015). The findings also indicated that substance abuse history and father's illiteracy increased the odds of alcohol consumption among street children. These findings suggest that families' low social class could increase their tendency toward alcohol consumption.

## 5 CONCLUSIONS

Street children in Iran reported lower rates of alcohol consumption compared to other countries. However, such a rate seems higher among street children than in Iran's general adolescent population. This rate could probably be high in some cities. However, planned programs for street children in Iran overlooked alcohol consumption, and some merely focused on the abuse of substances other than alcohol. The present study demonstrated that preventive plans for alcohol use among street children must be addressed by predicted services provided by governmental and nongovernmental organizations.

There are several limitations to note in this study. First, we only studied street children in some regions of Iran, and the sample size was small in the cities. Given cultural diversity and the impact of ethnic and religious contexts on peoples' tendency (including street children) toward alcohol consumption, different results may be obtained in other cities of Iran. Therefore, we recommend that future researchers conduct similar studies in other cities using a larger sample size and focus on different characteristics of children.

## COMPETING INTERESTS

The authors declare that they have no competing interests.

## AUTHOR CONTRIBUTIONS

All authors contributed substantially to text revision and approved the final manuscript.

## ETHICS APPROVAL AND CONSENT TO PARTICIPATE

The research procedure was approved by the Ethical Review Board of the University of Social Welfare and Rehabilitation Sciences (ethics approval number CODE IR.USWR.REC.1395.373). All participants provided verbal consent and answered the questionnaire anonymously.

## CONSENT FOR PUBLICATION

Not applicable

## AVAILABILITY OF DATA AND MATERIALS

Data are available upon reasonable request.

### PEER REVIEW

The peer review history for this article is available at: https://publons.com/publon/10.1002/brb3.2781
